# Two-Step Generation of Oligodendrocyte Progenitor Cells From Mouse Fibroblasts for Spinal Cord Injury

**DOI:** 10.3389/fncel.2018.00198

**Published:** 2018-07-25

**Authors:** Yukyeong Lee, C-Yoon Kim, Hye Jeong Lee, Jae Gon Kim, Dong Wook Han, Kisung Ko, James Walter, Hyung-Min Chung, Hans R. Schöler, Young Min Bae, Kinarm Ko

**Affiliations:** ^1^Department of Stem Cell Biology, School of Medicine, Konkuk University, Seoul, South Korea; ^2^Center for Stem Cell Research, Institute of Advanced Biomedical Science, Konkuk University, Seoul, South Korea; ^3^Department of Physiology, School of Medicine, Konkuk University, Seoul, South Korea; ^4^Department of Medicine, College of Medicine, Chung-Ang University, Seoul, South Korea; ^5^Research Service, Hines Veterans Administration Hospital, Hines, IL, United States; ^6^Department of Cell and Developmental Biology, Max Planck Institute for Molecular Biomedicine, Münster, Germany; ^7^Medical Faculty, University of Münster, Münster, Germany; ^8^Research Institute of Medical Science, Konkuk University, Seoul, South Korea

**Keywords:** oligodendrocyte progenitor cells, oligodendrocytes, neural stem cells, direct-converted neural stem cells, spinal cord injury

## Abstract

Oligodendrocyte progenitor cells (OPCs) are attracting attention as the ideal cell therapy for spinal cord injury (SCI). Recently, advanced reprogramming and differentiation techniques have made it possible to generate therapeutic cells for treating SCI. In the present study, we used directly-induced neural stem cells (DNSCs) from fibroblasts to establish OPCs (DN-OPCs) capable of proliferation and confirmed their OPC-specific characteristics. Also, we evaluated the effect of transplanted DN-OPCs on SCI in rats. The DN-OPCs exhibited an OPC-specific phenotype and electrophysiological function and could be differentiated into oligodendrocytes. In the SCI model, transplanted DN-OPCs improved behavior recovery, and showed engraftment into the host spinal cord with expression of myelin basic protein. These results suggest that DN-OPCs could be a new source of potentially useful cells for treating SCI.

## Introduction

Oligodendrocyte progenitor cells (OPCs) are a subtype of glial cells found in the central nervous system. OPCs originate in the neuroepithelium of the spine and differentiate through a pre-myelinating stage to become mature myelinating cells. Also, proliferating OPCs exist in through all the brain parenchyma (Menn et al., 2006) and these cells generate the myelin internode and thereby interact with axons to organize the nodal, paranodal and juxtaparanodal regions of myelinated axons (Bercury and Macklin, 2015).

Demyelination is the loss of the myelin sheath insulating nerves and occurs in response to spinal cord injury (SCI). This results in patients suffering from partial or complete loss of nervous system function (Finnerup et al., 2003). Remyelination is the process of propagating OPCs to form oligodendrocytes (OLs) to create new myelin sheaths on demyelinated axons, and regarded as essential progress for recovery from SCI (Finnerup et al., 2003).

Stem cell transplantation has attracted most attention as a promising therapeutic strategy for SCI (Dalamagkas et al., 2018). Previous studies showed that induced pluripotent stem cells (iPSCs)-derived OPCs enhanced functional recovery in a model for SCI (Kawabata et al., 2016). Although iPSCs are regarded as an excellent cell source without ethical concern or immune rejection, there is a concern for the safety of cell transplantation therapy due to tumorigenic potentials of iPSCs (Nishimori et al., 2014). Thus, there is a need for OPCs that continuously proliferate stably and are capable of minimizing the risk of tumorigenesis.

The ultimate goal of cell therapy in SCI is to ensure that the transplanted cells are stably implanted at the site of injury and lead to the development of OPCs that produce functional recovery by forming the myelin sheath (Miron et al., 2011). In this study, directly-induced neural stem cell (DNSC)-derived OPCs (DN-OPCs) were generated to produce a cell line capable of continuous proliferation and a high differentiation rate into OLs. In addition, we assessed the effects of transplanting DN-OPCs into an animal model of SCI. The results suggest the possibility of a new cell therapy for SCI.

## Materials and Methods

### Generation of OPCs From DNSCs

To convert DNSCs into OPC like cells, we used DNSCs generated previously (Han et al., 2012). DNSCs were seed at 1.5 × 10^5^ cells on matrigel (Corning, Corning, NY, USA) coated 24 well plate. For generation of OPCs, DNSCs were cultured for 3 days in OPC culture medium consisting of DMEM/F12 (Corning) with N2 supplement (Gibco, Waltham, MA, USA), B27 supplement without vitamin (Gibco), penicillin/streptomycin (Gibco), non-essential amino acid (Gibco), β-mercaptoethanol (Gibco), fibroblast growth factor (Peprotech, Seoul, Korea, 20 ng/ml), and platelet-derived growth factor-AA (Prospec, East Brunswick, NJ, USA, 20 ng/ml). OPC medium was freshly replaced every day. On the 3rd day, the cells were detached by 0.25% Trypsin-EDTA and re-plated on matrigel-coated 24 well plate in OPC culture medium for establishment of OPCs.

### Cell Counting

The number of cells was counted as an accumulative number for each subculture using an automated cell counter (Bio-Rad, Hercules, CA, USA) and counter slides (Bio-Rad) with a trypan blue (Sigma Aldrich, St. Louis, MO, USA). The numbers in the graphs were conceived through carefully checking them triple times.

### *In Vitro* Differentiation of OPCs Into OLs

OPCs were seed at 5 × 10^4^ cells on matrigel-coated 24 well plates. The next day, the cells were feed with OL differentiation medium including neural basal with B27 supplement without vitamin (Gibco), glutamax TM-1 (Gibco) and triiodothyronine (Sigma Aldrich, 30 ng/ml) for 3 days.

### Realtime PCR

To extract total RNA, we used a RNeasy Kit (Qiagen, Germany) following the supplier’s instructions. Total RNA (1 μg) was reverse-transcribed into cDNA using an Omniscript RT Kit (Qiagen) following the manufacture’s protocol. PCR reactions used Ex Taq polymerase (TaKaRa, Japan) and were performed for 25–28 cycles for all markers. Gene expression levels were evaluated by quantitative RT-PCR using SYBR Green (Thermo Scientific, Waltham, MA, USA) and a Roche real-time PCR system (Roche, Switzerland). Primer sequences used to amplify cDNA samples are listed in Supplementary Tables S1, S2.

### Immunocytochemistry

Cells were fixed in 4% paraformaldehyde for 10 min at room temperature and washed with dulbecco’s phosphate buffered saline (DPBS; Hyclone, Logan, UT, USA). For permeabilization, 0.5% Triton X-100 (Sigma Aldrich) in DPBS was added on fixed cells for 10 min at room temperature. The cells were blocked with 2% of bovine serum albumin (BSA; Gibco) in DPBS for 1 h at room temperature. Then, the cells were incubated in primary antibody solution overnight at 4°C. After washing with 0.2% tween 20 in DPBS, the cells were incubated in secondary antibody for 1 h at room temperature and washed with 0.2% tween 20 in DPBS. For nucleic acid staining, the cells were incubated in 4’-6-Diamidino-2-phenylindole (DAPI) for 5 min at room temperature and washed with 0.2% tween 20 in DPBS. Information of antibodies are listed in Supplementary Table S3 and Supplementary Figure S6.

### Electrophysiology

Whole-cell patch clamping for measuring ion channel currents of DNSCs and DN-OPCs (Passage 13) was performed within 2 days of attachment on coverslips (Knittel Glass, Germany) in culture medium. Potassium currents were recorded under the conventional whole-cell patch-clamp configuration. An Axopatch 200B patch-clamp amplifier and a Digidata 1550B interface (Axon Instruments, Union City, CA, USA) were used for voltage-clamp and data acquisition, respectively. Potassium current data were digitized using pClamp 10.6 software (Axon Instruments) at a sampling rate of 10 kHz, low-pass filtered at 1 kHz, and stored on a computer. The patch pipettes were pulled from borosilicate capillaries (Clark Electromedical Instruments, UK) using a puller (PP-83; Narishige, Japan). We used patch pipettes with a resistance of 2–3 MΩ when filled with pipette solutions. All experiments were carried out at room temperature (20–25°C). The cells for recording neural signals in patch clamp setting were continuously superfused with normal tyrode (NT) solution [78.4 mM NaCl (143 mM NaCl for outward K^+^ currents), 78.4 mM KCl (5.4 mM KCl for outward K^+^ currents), 0.33 mM NaH_2_PO_4_, 1.8 mM CaCl_2_, 0.5 mM MgCl_2_, 5 mM 4-(2-hydroxyethyl)-1-piperazineethanesulfonic acid (HEPES), and 11 mM glucose, adjusted to pH 7.4 with NaOH] used as the bathing solution. The pipette solution for recording K^+^ current contained 135 mM KCl, 5 mM NaCl, 10 mM HEPES, 5 mM EGTA, 10 μM 4, 4’-diisothiocyano-2, 2’-stilbenedisulfonic acid and 5 mM Mg-ATP; pH was adjusted to 7.2 with KOH. All chemicals and drugs were purchased from Sigma Aldrich. Barium chloride dihydrate (BaCl_2,_ 100 μM, inwardly rectifying K^+^ channel blocker, Sigma Aldrich) was prepared as stock solutions in distilled water. 4-aminopyridin (4-AP, 1 mM, voltage-dependent K^+^ channel blocker, Sigma Aldrich) was prepared as stock solutions in distilled water (pH 7.4 with HCl).

### Cell Staining (PKH26GL)

To track transplanted OPCs, PKH26GL Red fluorescent cell linker kit (Sigma Aldrich) was used for cell labeling. For staining, PKH26GL (PKH26GL 2 μl/diluent C buffer 500 μl) was added to OPCs suspended in diluent C buffer.

### Spinal Cord Contusion and Treatment

The experiment was carried out in accordance with animal ethics committee guidelines and approved by the Institutional Animal Care and Use Committee of the Konkuk University. Female Sprague–Dawley rats (250–280 g, Young bio, South Korea) were anesthetized using zoletil and xylazine (3:1 ratio, 1 ml/kg). Spinal laminectomy was performed at T9 site. Briefly, the muscles overlying the vertebral column were reflected, exposing the vertebral column T8–T10; the T9 spine segment was carefully removed. A 50-g clip-compression injury was performed at the T9. Without interruption of the dura mater or damage to adjacent dorsal and ventral roots, the clip was closed around the cord for 20 s 1 day after the operation, the experimental group (*n* = 10) was anesthetized and treated with DN-OPCs (1 × 10^6^ cells in 20 μl condition medium) implanted into the surgical site, whereas the control group was treated with 20 μl of PBS. Briefly, DN-OPCs and PBS were injected along the midline of the spinal cord at a depth of about 1.0 mm into the lesion epicenter using 31 g insulin syringes (BD., Franklin Lakes, NJ, USA). Then, the muscle and fascia were sutured and the skin was closed. Dextrose 2.5% half saline (20 ml/kg, Haflsol, Daehan, South Korea) was administered daily via tail vein catheter. The urinary bladder was emptied by abdominal compression at least twice daily until spontaneous partial voiding of the bladder occurred, usually after the first week.

### Behavioral Assessment

The behavioral recovery was evaluated by the Basso Beattie Bresnahan (BBB) open field locomotor test (Basso et al., 1995), for which the rats were placed in the middle of a square for 5 min. The BBB score was assigned by the scoring of behaviors including the trunk, tail, and hind limbs. Two independent observers evaluated the spinal cord sensory motor function of the animals after SCI. Before testing, the animals’ bladders were expressed because spontaneous bladder contraction often accompanies hind limb activity. Functional recovery was analyzed twice a week for up to 4 weeks using BBB score analysis.

### Immunohistochemistry

All rats were sacrificed at 5 weeks after operation. The spinal cord was removed and fixed with 4% paraformaldehyde. During autopsy the spinal cord above and below the level of injury was observed and the diameter measured to determine if atrophy was present. Tissue samples were rinsed with PBS and stored in PBS with 30% sucrose overnight. Tissue samples were embedded in optimal cutting temperature (OCT) compound (Sakura Finetek, Japan) media; 10 μm longitudinal sections were obtained. The sections were incubated with the primary antibody: anti-neurofilament 200 (NF200; Sigma Aldrich, St. Louis, MO, USA), anti-glial fibrillary acidic protein (GFAP; Chemicon, Temecula, CA, USA), anti-myelin basic protein (MBP; Biolegend, San Diego, CA, USA) for 72 h at 4°C. The sections were then washed with PBS and incubated with Alexa 488 or 594 conjugated secondary antibody (Molecular Probes, Cambridge, MA, USA) for 1 h at room temperature and mounted with DAPI. Each evaluation proceeded at the interface of the injured area of the spinal cord, GFAP (an astrocytic scar-related marker) in the gray matter, and NF200, MBP in the white matter. For each sample, three images of the injured area were randomly selected. Images were acquired using a fluorescence microscope fitted with a digital camera system (Nikon, Japan), and routed to a Windows PC for quantitative analyses using Adobe Photoshop CS5 software. Attention was given to ensure identical settings for fluorescence exposure, amplifier gain and cut off across all images. Information of antibodies are listed in Supplementary Table S4.

### Statistics

Statistical analyses were conducted using the Statistical Analysis System software (SAS; SAS Korea, Inc., South Korea), version Enterprise 4.0. Data are presented as the means ± SEM. All statistics were calculated using independent *t*-tests or analysis of variance (ANOVA) with least significant differences (LSD) tests for *post hoc* analysis. A value of *p* < 0.05 was considered significant.

## Results

### Two-Step Generation of OPCs From NSCs

Before generating DN-OPCs, we first derived OPCs from forebrain-derived NSCs (FNSCs) to ensure stable induction of OPCs from NSCs. OPCs derived from FNSCs (FN-OPCs) stand out as a typical feature of OPCs, a bipolar and tripolar morphology (Supplementary Figure S1). Also, we confirmed that FN-OPCs expressed OPC-specific markers and increased expression of OPC-specific genes (Supplementary Figures S3, S4). In addition, the number of cells per passage was measured to verify that FN-OPCs were able to proliferate continuously (Supplementary Figure S2).

**Figure 1:**
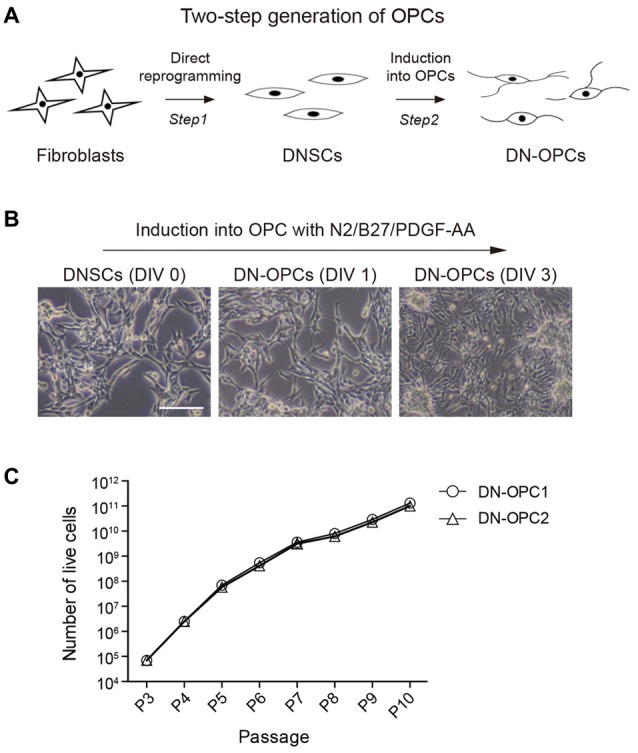
Two-step generation of oligodendrocyte progenitor cells (OPCs) from neural stem cells (NSCs). **(A)** Diagram of experiment. The process of generating of direct conversion directly-induced neural stem cells (DNSCs) derived OPCs (DN-OPCs). **(B)** DNSC-derived OPCs Induction. DNSCs have a capacity of differentiation into OPCs. **(C)** Growth curves of DN-OPCs by the increase in passage. Data are present as the means ± SEM (*n* = 3). Scale bar: 250 μm.

**Figure 2:**
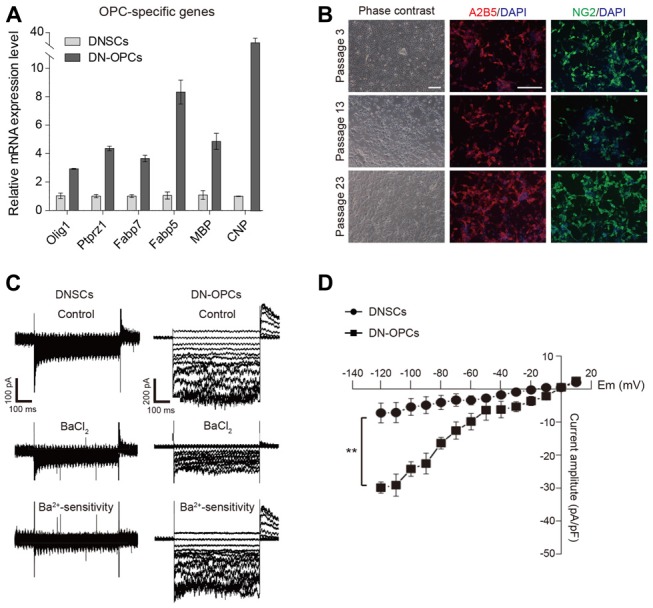
Analysis of molecular and cellular characteristics of OPCs. **(A)** Quantitative RT-PCR analysis. OPCs related mRNA expression level in DN-OPCs relative to DNSCs. Graphs represent changes after normalization to NSCs. **(B)** Consistent morphology. Consistent morphology of OPCs at early (P3), middle (P13) and late (P23) passage. DN-OPCs can maintain steady morphology despite increase in passage. Immunofluorescence images of OPCs stained with OPC specific markers (A2B5 and NG2) at early and late passage. Cells were counterstained with DAPI. **(C)** Patch-clamp analysis of DNSCs and DN-OPCs. Inward K^+^ currents in DNSCs and DN-OPCs, and the response to Ba^2+^. Ba^2+^-sensitive currents. **(D)** Curve of currents sensitive to Ba^2+^. Data are presented as the means ± SEM (*n* = 3). Scale bar: 250 μm. **p < 0.005.

Using the established method for generation of OPCs from NSCs, we induced OPCs from DNSCs. The process of generating of DN-OPCs from DNSCs is summarized in Figure 1A. During the induction process of DN-OPCs, morphological changes in DNSCs first appeared within 3 days (Figure 1B). Through cell proliferation growth curves, we assessed the self-renewal capacity of OPCs (Figure 1C). The number of DN-OPCs increased continuously as the passage number increased. mRNA of OPC-specific genes was highly expressed in DN-OPCs (Passage 13) in comparison to DNSCs (Figure 2A). We also checked OPC-specific markers (A2B5 and NG2) in DN-OPCs (Passage 3, Passage 13 and Passage 23) at protein level (Figure 2B). These results indicate that DN-OPCs established from DNSCs are stably proliferative and can maintain the characteristics of OPCs.

Voltage-gated K^+^ (outward K^+^, Kv) currents and inward K^+^ (Kir) have been detected in OPCs (Soliven et al., 1988; Jiang et al., 2013). To verify that DN-OPCs have an electrophysiological characteristic of typical OPCs, we measured the voltage-dependence of K^+^ currents. To investigate whether DN-OPCs possessed Kir currents, whole-cell patch clamping of DNSCs and DN-OPCs (Passage 13) was carried out (Figure 2C). Kir currents were elicited using 500-ms-long depolarizing voltage step pulses from −120 mV to +10 mV from the holding potential of 0 mV. We measured the voltage of activation of Kir currents in DN-OPCs. As shown in Figure 2C, Kir currents were detected in DN-OPCs. Furthermore, application of Ba^2+^ blocked Kir currents in DN-OPCs. On the other hand, there was no effect on DNSCs Kir currents after the application of Ba^2+^. Ba^2+^-sensitive K^+^ currents measured in both groups are shown in bottom panels of Figure 2C. We found that there was almost no Ba^2+^-sensitive current in DNSCs, but large Ba^2+^-sensitive currents were recorded in DN-OPCs (Figure 2D).

### Efficiency of Differentiation Into Oligodendrocyte

**Figure 3:**
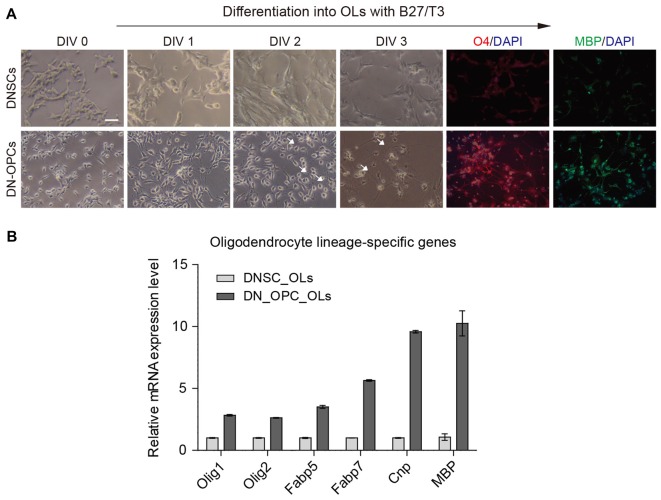
Efficiency of differentiation into oligodendrocytes (OLs). **(A)** Difference of morphology during differentiation into OLs and immunocytochemistry. **(B)** Quantitative RT-PCR analysis. Glial related mRNA expression level between DNSC-derived OLs and DN-OPC-derived OLs. Graphs represent changes after normalization to DNSC-derived OLs. Data are present as the means ± SEM (*n* = 3). Scale bar: 250 μm. The white arrows indicate multiple branches.

As precursor cells, OPCs can differentiate more effectively than NSCs, both in terms of differentiation potency into OLs and differentiation time. Thus, DN-OPCs are expected to play a role as precursors of glial cells that carry signals from nerves. In this experiment, we induced differentiation into OLs from DNSCs and DN-OPCs during the same period (3 days). As shown in Figure 3A, morphologic changes to multipolar cells, thin process, and multiple branches (white arrows), were observed more quickly in DN-OPCs than DNSCs. OL maker genes (O4 and MBP) were more clearly expressed in OLs differentiated from DN-OPCs than them from DNSCs. We also found that DN-OPC-derived OLs showed higher expression of OL-related genes (*Olig1*, *Olig2*, *Fabp5*, *Fabp7*, *Cnp* and *Mbp*) compared with DNSC-derived OLs (Figure 3B). These results indicate that DN-OPCs are more efficient cell source for differentiation into OLs than NSCs.

### Behavioral Assessment After Transplantation

**Figure 4:**
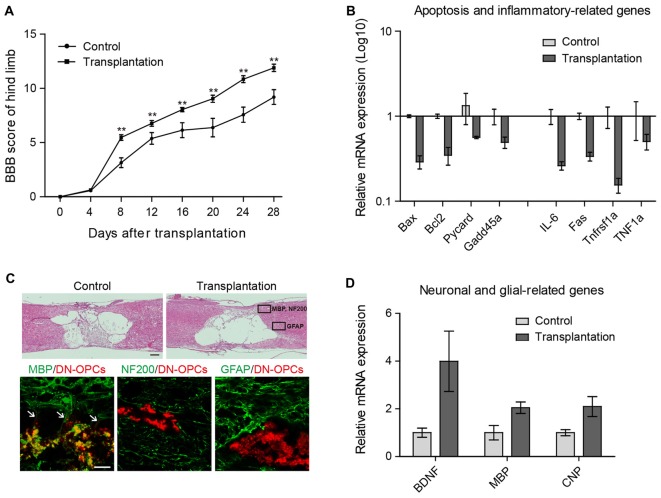
Functional recovery after transplantation. **(A)** Basso beattie bresnahan (BBB) Score. The Mean BBB motor score for hind limb in control and DN-OPCs transplantation groups after spinal cord injury (SCI) surgery. **(B)** Quantitative RT-PCR analysis. Apoptosis and inflammation related genes in spinal cord. Graphs represent changes after normalization to control. **(C)** H&E Staining of control and DN-OPCs transplantation groups and immunohistochemical analysis of DN-OPCs transplantation groups. Myelin basic protein (MBP) is indicated with white arrows **(D)** Quantitative RT-PCR analysis. Brain-derived neurotrophic factor (BDNF), MBP and CNP in spinal cord. Graphs represent changes after normalization to control. Data are presented as the means ± SEM (*n* = 3). Scale bar: 500 μm. **p < 0.001.

To validate *in vivo* functionality of DN-OPCs, DN-OPCs were transplanted into our SCI model rat. The BBB scores of DN-OPC transplanted animals were compared with control animals for 28 days twice a week (Figure 4A). Four days after surgery, the BBB scores of the DN-OPCs group were significantly higher than that of the controls over the entire duration of the behavioral analysis. Motor recovery in the control group was poor after 1 week, whilst the DN-OPCs group showed continuous restoration of motor function for 4 weeks. The BBB score of the DN-OPCs group at 28 days was 11.89 ± 0.51, significantly different from controls (8.82 ± 0.86; ANOVA, *p* < 0.01; Figure 4).

On the basis of the BBB scores, we evaluated differences in the expression of genes related to apoptosis and inflammation. There was a 2-fold increase in the expression of apoptosis-related gene mRNA (*Bax*, *Bcl-2, Pycard* and *Gadd45a*) in the DN-OPCs compared to the control group (Figure 4B). Expression of *IL-6*, *Fas*, *Tnfrsf1a* and *TNF1α*, a known inflammation-related gene, dropped by half or more in the DN-OPCs transplantation group compared to the control group (Figure 4B). These results demonstrated that DN-OPCs transplantation has resulted in behavioral recovery with inflammation control in SCI model.

### Increase in Differentiation Efficiency of DN-OPCs Into Oligodendrocytes After Transplantation

Spinal cord tissues were isolated 4 weeks after surgery for histological analysis, and the tissue sections were examined for the presence and differentiation of DN-OPCs. In the mid-line parasagittal areas of hematoxylin and eosin stained sections, each group contained large cysts in the spinal cord formed by inflammation after contusion, and there was no significant difference between the two groups (Figure 4C).

Neural fates of the transplanted cells were identified using specific markers for each cell type (astrocytes: GFAP; neurons: NF200; OLs: MBP). Differentiation was assessed in the marked areas of Figure 4C considering the intact distribution according to each cell type. As shown in Figure 4C, GFAP or NF200 were not expressed in transplanted DN-OPCs. In addition, no integration with surrounding GFAP- or NF200-positive cells was observed. In contrast, MBP was strongly expressed in the transplanted cells. We found that neurites were projected from the transplanted cells integrated with surrounding cells (Figure 4C). Analysis of mRNA expression of neuronal and glial-related genes showed that expression of brain-derived neurotrophic factor and glial (BDNF), MBP, and CNP in the DN-OPCs transplantation group was higher than the control group (Figure 4D). These results show that transplanted DN-OPCs were clearly differentiated into OLs and were integrated into the surrounding OLs of the recipient.

## Discussion

The purpose of this study was to establish method of generation of proliferative OPCs from DNSCs. Following the induction of DN-OPCs, we compared them with NSCs in terms of morphology and gene expression level and confirmed the specific morphology of DN-OPCs. The expression of OPC-related gene mRNA was up-regulated in DN-OPCs relative to DNSCs. We also confirmed the expression of OPC-specific marker genes at early, middle and late passages. These results support that our OPCs generated from DNSCs are stable cell lines which are proliferative and maintain OPC characteristics, suggesting the possibility of a more useful source of therapeutic cells for SCI.

It has been reported that potassium channels were upregulated in OPCs, and OPCs were able to efficiently detect potassium increases generated by an action potential (Maldonado et al., 2013). We confirmed that inwardly rectifying channel for potassium of DN-OPCs was amplified compared to DNSCs. Furthermore, application of Ba^2+^ blocked Kir only in DN-OPCs, but not in DNSCs. Also, Ba^2+^-sensitive current amplitude (pA/pF) was profoundly different in DNSCs vs. DN-OPCs. Only DN-OPCs were sensitive to Ba^2+^. Additionally, we measured outward K^+^ (Kv) currents to confirm that DNSCs and DN-OPCs were electrophysiologically different. Kv currents were upregulated in DN-OPCs and the inhibitory effect of 4-AP was more apparent in DN-OPCs than NSCs (Supplementary Figure S5). These data demonstrate that differentiated DN-OPCs are more closely related to OLs than DNSCs functionally, and DNSCs and DN-OPCs are electrophysiologically different cells.

Once the cells are differentiated into OLs, they have a thin process and form multiple branches (Pedraza et al., 2014). To identify the efficiency of differentiation into OLs from DNSCs and DN-OPCs, we confirmed that DN-OPCs showed faster and more distinct OL-specific morphology and gene expression than DNSCs during the same time period and the same differentiation environment. This result indicates that OPCs have a higher efficiency and differentiation ability than DNSCs. Thus, DN-OPCs are an ideal cell source for inducing differentiation into OLs.

In the BBB score analysis, the DN-OPCs group showed a significant recovery effect from the beginning (day 8) of recovery after SCI. The BBB score increased steadily up to 28 days. Long-term recovery is needed, but the animals were sacrificed 28 days after surgery to perform cell tracing and detect chronic inflammation in this study. The expression of pro-inflammatory genes and apoptotic factors was determined in the damaged area. The results showed that the DN-OPCs-treated group had a significant decrease in the expression of each gene, suggesting that recovery of the BBB score was due to a reduction in inflammation.

Tissue analysis was performed to determine whether the transplanted cells actually affected the damaged area. Unfortunately, unlike gene and behavioral analyses, the cysts produced by chronic inflammation were similar in both groups. This suggests that the effect of cell transplantation cannot overcome the tissue damage caused by SCI contusion. Rather, these results imply that transplanted cells can be adversely affected by the inflammation caused by SCI. This is an area that needs to be addressed in the future-the limitation of spinal cord cist and scar tissue formation. This is part of a consideration of the cell transplant approach using the current SCI model, along with previous cell transplantation studies. Despite the lack of effect on cyst formation, transplanted cells were observed at the boundary of the injured site, and we performed immunostaining to confirm engraftment of these cells. To minimize effects on the character of the cells, tracking using DiI labeling was performed without a viral integration system. Although the fluorescence image is a bit crude due to features of DiI appearing in the cytoplasm, it is useful for analyzing the results because cell tracing remains possible without nonspecific artifacts of staining.

As shown in Figure 4C, transplanted cells were observed in both the gray and white matter of the spinal cord, and staining was performed according to neuronal distribution (OLs, neurons and astrocytes). Interestingly, transplanted cells differentiated into OLs in accordance with the direction in which the differentiation was originally triggered and integrated with the surrounding host OLs. On the other hand, the staining of neurons and astrocytes showed a clearly distinct fraction isolated in the tissues and revealed no differentiation between neurons and astrocytes. In addition, genetic analysis of the site of injury revealed a relative increase in OL-specific marker gene expression. These results demonstrate that the DN-OPCs developed in this study differentiate after transplantation into the spinal cord as programmed, and function by integration into the environment of the host.

In conclusion, the results of this study demonstrated that DN-OPCs derived from DNSCs by growth factor composition can stably self-renew and efficiently differentiate into OLs. In addition, we confirmed the positive effect of DN-OPCs on functional recovery in a rat model of SCI *in vivo*. Therefore, this study suggests the possibility that DNSC-derived OPCs and OPC-derived OLs, could be useful cell sources to treat SCI.

## Author Contributions

YL and C-YK designed the conception and design of experiments, and performed the experiments and analyzed data together with HJL and JGK. YL wrote the manuscript. DWH, KisungKo, JW, H-MC, HS and YMB contributed to the reagents, materials, and analysis tools. KinarmKo contributed to the conception and design of experiments, financial support, manuscript writing and final approval of the manuscript.

## Conflict of Interest Statement

The authors declare that the research was conducted in the absence of any commercial or financial relationships that could be construed as a potential conflict of interest.

## References

[B1] Basso D. M. Beattie M. S. Bresnahan J. C. (1995). A sensitive and reliable locomotor rating scale for open field testing in rats. J. Neurotrauma 12, 1–21. 10.1089/neu.1995.12.1 7783230

[B2] Bercury K. K. Macklin W. B. (2015). Dynamics and mechanisms of CNS myelination. Dev. Cell 32, 447–458. 10.1016/j.devcel.2015.01.016 25710531 PMC6715306

[B3] Dalamagkas K. Tsintou M. Seifalian A. M. (2018). Stem cells for spinal cord injuries bearing translational potential. Neural Regen. Res. 13, 35–42. 10.4103/1673-5374.224360 29451202 PMC5840986

[B4] Finnerup N. B. Johannesen I. L. Fuglsang-Frederiksen A. Bach F. W. Jensen T. S. (2003). Sensory function in spinal cord injury patients with and without central pain. Brain 126, 57–70. 10.1093/brain/awg007 12477697

[B5] Han D. W. Tapia N. Hermann A. Hemmer K. Höing S. Araúzo-Bravo M. J.. (2012). Direct reprogramming of fibroblasts into neural stem cells by defined factors. Cell Stem Cell 10, 465–472. 10.1016/j.stem.2012.02.021 22445517

[B6] Jiang P. Chen C. Liu X. B. Selvaraj V. Liu W. Feldman D. H.. (2013). Generation and characterization of spiking and nonspiking oligodendroglial progenitor cells from embryonic stem cells. Stem Cells 31, 2620–2631. 10.1002/stem.1515 23940003 PMC3923867

[B7] Kawabata S. Takano M. Numasawa-Kuroiwa Y. Itakura G. Kobayashi Y. Nishiyama Y.. (2016). Grafted human iPS cell-derived oligodendrocyte precursor cells contribute to robust remyelination of demyelinated axons after spinal cord injury. Stem Cell Reports 6, 1–8. 10.1016/j.stemcr.2015.11.013 26724902 PMC4719132

[B8] Maldonado P. P. Vélez-Fort M. Levavasseur F. Angulo M. C. (2013). Oligodendrocyte precursor cells are accurate sensors of local K^+^ in mature gray matter. J. Neurosci. 33, 2432–2442. 10.1523/JNEUROSCI.1961-12.2013 23392672 PMC6619152

[B9] Menn B. Garcia-Verdugo J. M. Yaschine C. Gonzalez-Perez O. Rowitch D. Alvarez-Buylla A. (2006). Origin of oligodendrocytes in the subventricular zone of the adult brain. J. Neurosci. 26, 7907–7918. 10.1523/JNEUROSCI.1299-06.2006 16870736 PMC6674207

[B10] Miron V. E. Kuhlmann T. Antel J. P. (2011). Cells of the oligodendroglial lineage, myelination, and remyelination. Biochim. Biophys. Acta 1812, 184–193. 10.1016/j.bbadis.2010.09.010 20887785

[B11] Nishimori M. Yakushiji H. Mori M. Miyamoto T. Yaguchi T. Ohno S.. (2014). Tumorigenesis in cells derived from induced pluripotent stem cells. Hum. Cell 27, 29–35. 10.1007/s13577-013-0078-3 24122447

[B21] Pedraza C. E. Taylor C. Pereira A. Seng M. Tham C. S. Izrael M.. (2014). Induction of oligodendrocyte differentiation and *in vitro* myelination by inhibition of rho-associated kinase. ASN Neuro. 6:1759091414538134. 10.1177/1759091414538134 25289646 PMC4189421

[B14] Soliven B. Szuchet S. Arnason B. G. Nelson D. J. (1988). Voltage-gated potassium currents in cultured ovine oligodendrocytes. J. Neurosci. 8, 2131–2141. 10.1523/JNEUROSCI.08-06-02131.1988 2838593 PMC6569331

