# Supplementary material for: Two-Step Generation of Oligodendrocyte Progenitor Cells From Mouse Fibroblasts for Spinal Cord Injury
Source: Front Cell Neurosci. 2018 Jul 25;12:198. doi: 10.3389/fncel.2018.00198 (PMC6070016; doi:10.3389/fncel.2018.00198)
Supplement: Supplementary file 1 [file Table_1.DOCX]

**Supplementary Table 1. Primer sequences for PCR**

| Gene | Sequences (5′-3′) | Usage |
| --- | --- | --- |
| *Olig1* | F-TAT GAG CTG GTG GGT TAC AGG | qRT-PCR |
|  | R-CTG CTG CTG TTC CTC TTT GG |  |
| *Olig2* | F-GAC AAG AAG CAG ATG ACT GAG C | qRT-PCR |
|  | R-TGG CGA TGT TGA GGT CGT |  |
| *Ptprz1* | F-TTC ACT CCA TCC TCC AGA CC | qRT-PCR |
|  | R-ATG CAA GGC CGA ATC ACT AC |  |
| *Fabp5* | F-CAA AAC CGA GAG CAC AGT GA | qRT-PCR |
|  | R-CCC TCA TTG CAC CTT CTC AT |  |
| *Fabp7* | F-GGG TAA GAC CCG AGT TCC TC | qRT-PCR |
|  | R-ATC ACC ACT TTG CCA CCT TC |  |
| *Mbp* | F-GAG AAC TAC CCA TTA TGG CT | qRT-PCR |
|  | R-GGT GTT CGA GGT GTC ACA AT |  |
| *Cnp* | F-GCC ACT CTA CTT TGG CTG GT | qRT-PCR |
|  | R-GCA CAC CTG GAG GTC TCT TT |  |
| *β-actin* | F-CGT GCG TGA CAT CAA AGA GAA GC | qRT-PCR |
|  | R-ATC TGC TGG AAG GTG GAC AGT GAG |  |

qRT-PCR, quantitative RT-PCR; F, forward primer; R, reverse primer
