# Supplementary material for: Two-Step Generation of Oligodendrocyte Progenitor Cells From Mouse Fibroblasts for Spinal Cord Injury
Source: Front Cell Neurosci. 2018 Jul 25;12:198. doi: 10.3389/fncel.2018.00198 (PMC6070016; doi:10.3389/fncel.2018.00198)
Supplement: Supplementary file 2 [file Table_2.DOCX]

**Supplementary Table 2. Primer sequences for PCR**

| Gene | Sequences (5′-3′) | Usage |
| --- | --- | --- |
| *Bax* | F-CAC ATG GCA GAC AGT GAC CAT C | qRT-PCR |
|  | R-GGC CTC AGC CCA TCT TCT TC |  |
| *Bcl2* | F-CCA GCA TGC GAC CTC TGT TT | qRT-PCR |
|  | R-CAC TTG TGG CCC AGG TGT GC |  |
| *Pycard* | F-ACC AGG CAG TTC GTG CAG AG | qRT-PCR |
|  | R-GCC TCA AGG CCT CAA GGA AC |  |
| *Gadd45a* | F-AGG AGG AAG CTG TGT GGA AA | qRT-PCR |
|  | R-GAG GGC ATG ASAG ACC AAA AA |  |
| *Il-6* | F-CCA ACT CAT CTT GAA AGC ACT TGA A | qRT-PCR |
|  | R-TGA CCA CAG TGA GGA ATG TCC A |  |
| *Fas* | F-TGG CTT AGT GAT TGC ATC TCG TT | qRT-PCR |
|  | R-CGC AGG GTC TCT GTC CTC CT |  |
| *Tnfrsf1a* | F-CTG GAG AAC ATC CGC GAG AC | qRT-PCR |
|  | R-TCG AGT CCC GTT CCT GAG GT |  |
| *Tnf1a* | F-ATT TGG TGA CCA GGC TGT CG | qRT-PCR |
|  | R-CCA AGC GAA CTT TAT TTC TCT CAA TG |  |
| *Bdnf* | F-GGA ACT CGC AAT GCC GAA CT | qRT-PCR |
|  | R-TGA ACC GCC AGC CAA TTC TC |  |
| *Mbp* | F-AAC ATT GTG ACA CCT CGT ACA | qRT-PCR |
|  | R-TGT CTC TTC CTC CCC AGC TA |  |
| *Cnp* | F-ATG CCC AAC AGG ATG TGG TG | qRT-PCR |
|  | R-AGA GGG CAG AGA TGG ACA GT |  |
| *β-actin* | F-AGG GAA ATC GTG CGT GAC | qRT-PCR |
|  | R-CGC TCA TTG CCG ATA GTG |  |

qRT-PCR, quantitative RT-PCR; F, forward primer; R, reverse primer
