# Supplementary material for: Two-Step Generation of Oligodendrocyte Progenitor Cells From Mouse Fibroblasts for Spinal Cord Injury
Source: Front Cell Neurosci. 2018 Jul 25;12:198. doi: 10.3389/fncel.2018.00198 (PMC6070016; doi:10.3389/fncel.2018.00198)
Supplement: Supplementary file 3 [file Table_3.DOCX]

**Supplementary Table 3. Antibodies for immunocytochemistry**

| Name | Source | Dilution |
| --- | --- | --- |
| A2B5 | Millipore (Temecula, MA, US) | 1:500 |
| NG2 | Millipore (Temecula) | 1:300 |
| O4 | Millipore (Billerica, MA, US) | 1:300 |
| MBP | Bioregend (San Diego, CA, US) | 1:500 |
| DAPI | Sigma-Aldrich (St. Louis, US) | 1:1000 |
| Anti-mouse Alexa 488 IgG | Cell Signaling Technologies | 1:1000 |
| Anti-mouse Alexa 568 IgM | Cell Signaling Technologies | 1:1000 |
