# Supplementary material for: Two-Step Generation of Oligodendrocyte Progenitor Cells From Mouse Fibroblasts for Spinal Cord Injury
Source: Front Cell Neurosci. 2018 Jul 25;12:198. doi: 10.3389/fncel.2018.00198 (PMC6070016; doi:10.3389/fncel.2018.00198)
Supplement: Supplementary file 4 [file Table_4.DOCX]

**Supplementary Table 4. Antibodies for immunohistochemistry**

| Name | Source | Dilution |
| --- | --- | --- |
| MBP | Biolegend (US) | 1:500 |
| NF200 | Sigma (US) | 1:100 |
| GFAP | Chemicon (US) | 1:1000 |
| Anti- mouse  Alexa 488 IgG | Molecular Probes (US) | 1:1000 |
| Anti-mouse  Alexa 594 IgG | Molecular Probes (US) | 1:1000 |
