# Supplementary material for: Two-Step Generation of Oligodendrocyte Progenitor Cells From Mouse Fibroblasts for Spinal Cord Injury
Source: Front Cell Neurosci. 2018 Jul 25;12:198. doi: 10.3389/fncel.2018.00198 (PMC6070016; doi:10.3389/fncel.2018.00198)
Supplement: Supplementary file 5 [file Image_1.pdf]

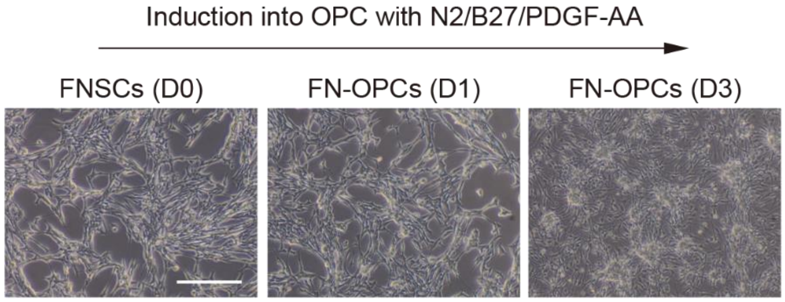

Supplementary Figure 1. Generation of FN-derived OPCs. Induction of forebrain neural stem cell-derived OPCs (FN-OPCs). Forebrain-NSCs have a capacity of differentiation into OPCs. Scale bar: 250 μm.
