# Supplementary material for: Two-Step Generation of Oligodendrocyte Progenitor Cells From Mouse Fibroblasts for Spinal Cord Injury
Source: Front Cell Neurosci. 2018 Jul 25;12:198. doi: 10.3389/fncel.2018.00198 (PMC6070016; doi:10.3389/fncel.2018.00198)
Supplement: Supplementary file 6 [file Image_2.pdf]

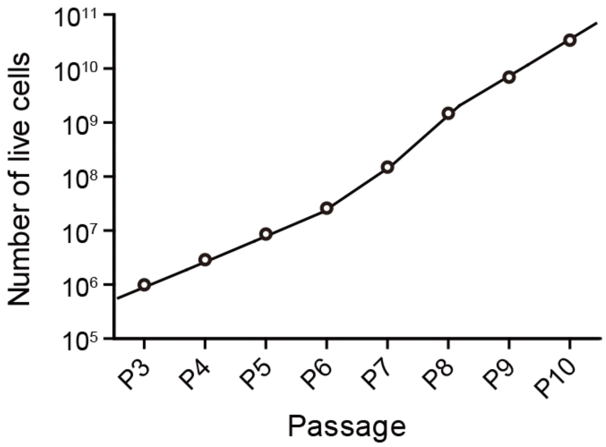

Supplementary Figure 2. Growth curve of FN-OPCs. Growth curve of FN-OPCs by the increase in passage. Data are present as the means ± SEM (n = 3).
