# Supplementary material for: Two-Step Generation of Oligodendrocyte Progenitor Cells From Mouse Fibroblasts for Spinal Cord Injury
Source: Front Cell Neurosci. 2018 Jul 25;12:198. doi: 10.3389/fncel.2018.00198 (PMC6070016; doi:10.3389/fncel.2018.00198)
Supplement: Supplementary file 7 [file Image_3.pdf]

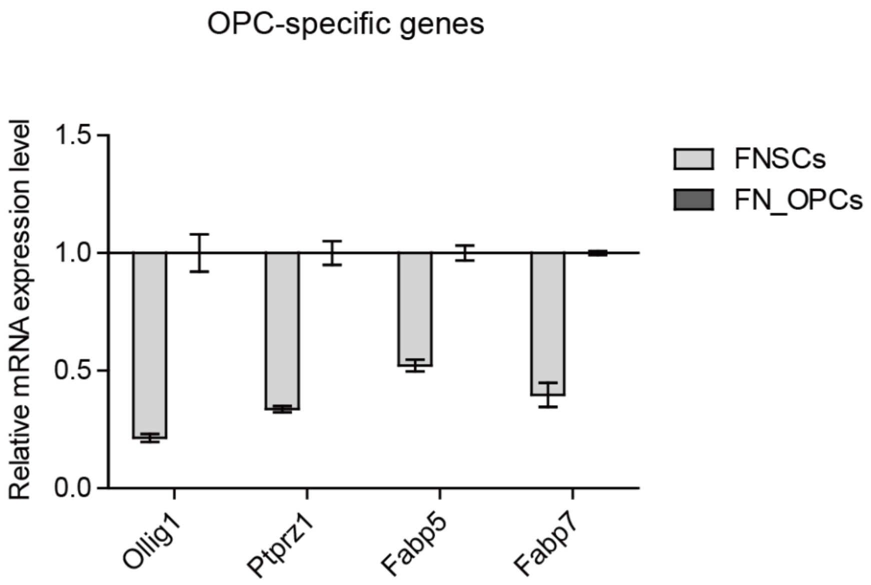

Supplementary Figure 3. OPCs related mRNA expression level in FNSCs and FN-OPCs. Quantitative RT-PCR analysis. OPC related mRNA gene expression level in FN-OPCs relative to NSC. Graphs represent changes after normalization to NSCs. Data are presented as means ± SEM (*n* = 3).
