# Supplementary material for: Two-Step Generation of Oligodendrocyte Progenitor Cells From Mouse Fibroblasts for Spinal Cord Injury
Source: Front Cell Neurosci. 2018 Jul 25;12:198. doi: 10.3389/fncel.2018.00198 (PMC6070016; doi:10.3389/fncel.2018.00198)
Supplement: Supplementary file 8 [file Image_4.pdf]

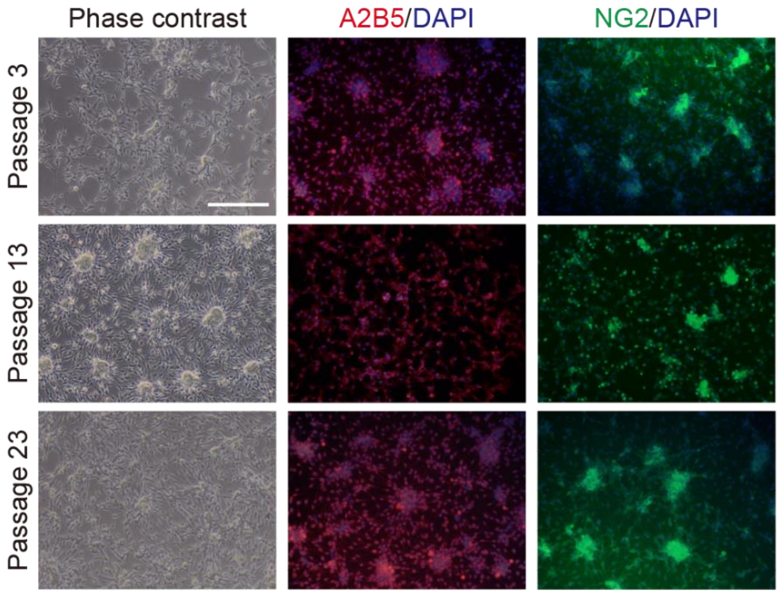

Supplementary Figure 4. OPC-specific markers at early, middle, and late passage. Consistent morphology. Consistent morphology of OPCs at early (P3), middle (P13), and late (P23) passage. DINSC derived OPC can maintain steady morphology despite increase in passage. Immunofluorescence images of OPCs stained with OPC specific markers (A2B5 and NG2) at early and late passage. Cells were counterstained with DAPI. Scale bar: 500 μm.
