# Supplementary material for: Two-Step Generation of Oligodendrocyte Progenitor Cells From Mouse Fibroblasts for Spinal Cord Injury
Source: Front Cell Neurosci. 2018 Jul 25;12:198. doi: 10.3389/fncel.2018.00198 (PMC6070016; doi:10.3389/fncel.2018.00198)
Supplement: Supplementary file 9 [file Image_5.pdf]

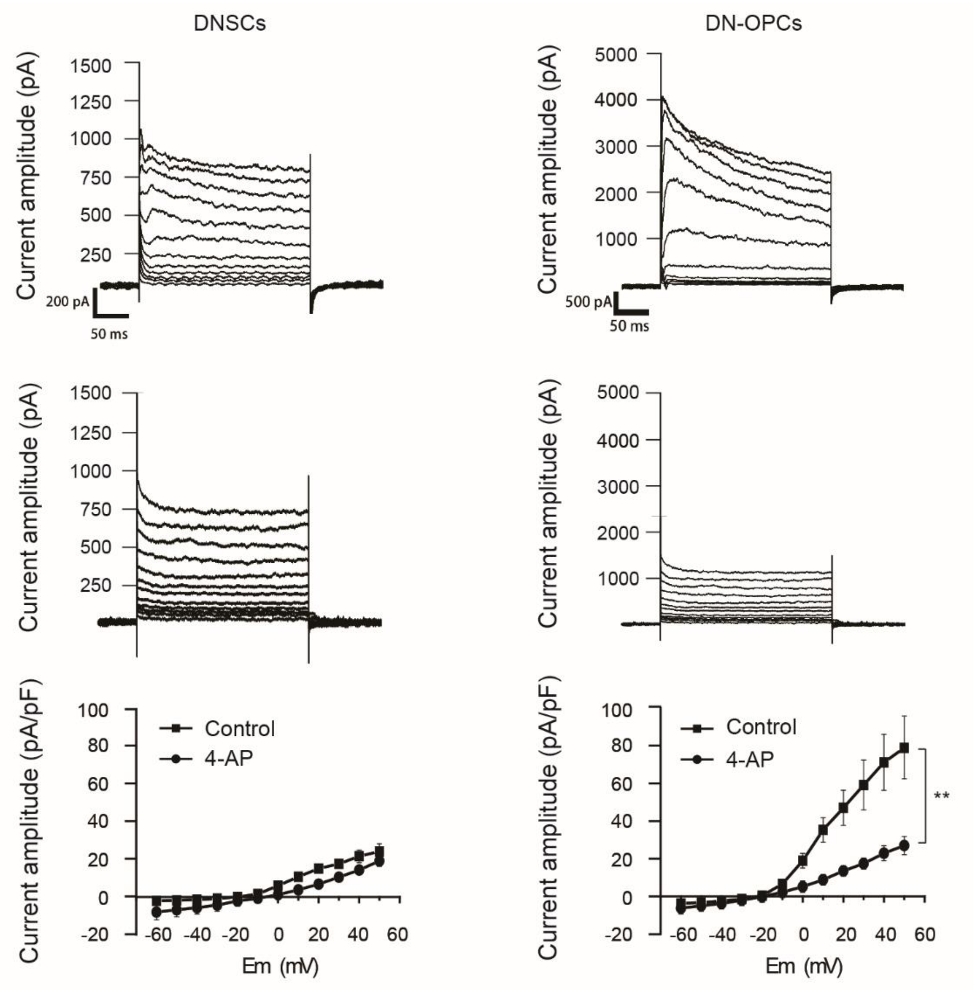

Supplementary Figure 5. Patch-clamp analysis of DNSCs and DN-OPCs. Outward K+ (Kv) currents in DNSCs and the response to 4-AP (left). Kv currents in DN-OPCs and the response to 4-AP (right). Current-voltage relationships of peak and steady current before and after application of 4-AP. Data are presented as means ± SEM (n = 3). **p < 0.001.
