# Supplementary material for: Two-Step Generation of Oligodendrocyte Progenitor Cells From Mouse Fibroblasts for Spinal Cord Injury
Source: Front Cell Neurosci. 2018 Jul 25;12:198. doi: 10.3389/fncel.2018.00198 (PMC6070016; doi:10.3389/fncel.2018.00198)
Supplement: Supplementary file 10 [file Image_6.pdf]

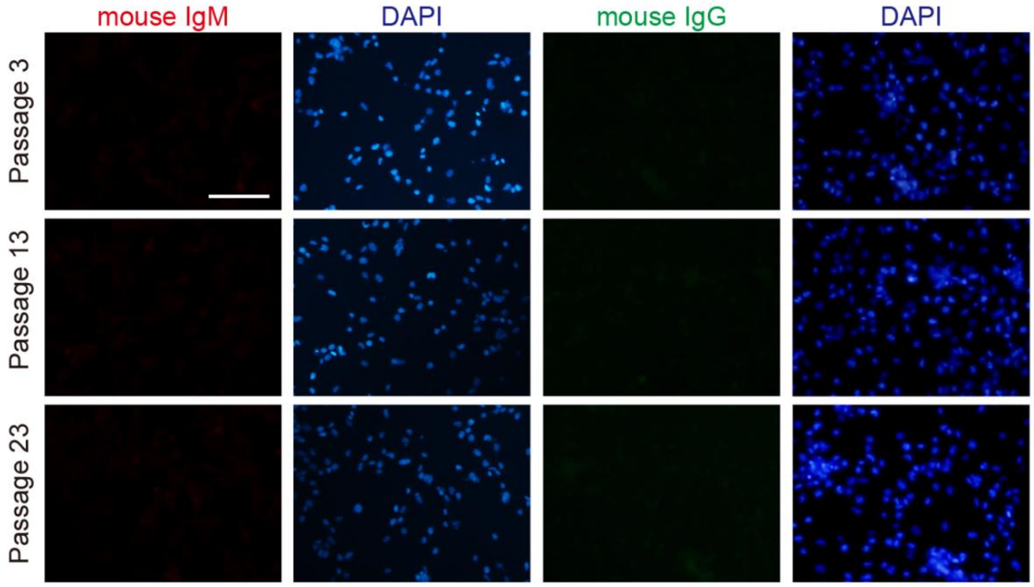

Supplementary Figure 6. Specificity of secondary antibody. Reaction of secondary antibody IgM (A2B5) and IgG (NG2) at early (P3), middle (P13), and late (P23) passage. Cells were counterstained with DAPI. Scale bar: 250 μm.
